# Population pharmacokinetics of methylprednisolone in neonates and paediatric patients undergoing cardiac surgery with cardiopulmonary bypass

**DOI:** 10.1007/s00228-026-04168-7

**Published:** 2026-08-18

**Authors:** Matias Rantanen, Juho Keski-Nisula, Klaus T. Olkkola, Pertti J. Neuvonen, Eero Pesonen, Pertti Suominen, Teijo I. Saari

**Affiliations:** 1 Department of Anaesthesiology and Intensive Care, University of Turku, Kiinamyllynkatu 4-8, Turku, 20521 Finland; 2 Division of Perioperative Services, Intensive Care and Pain Medicine, Turku University Hospital, Turku, Finland; 3 Department of Anaesthesiology and Intensive Care Medicine, University of Helsinki, Helsinki, Finland; 4 HUS Helsinki University Hospital, Helsinki, Finland; 5 Individualized Drug Therapy Research Program, Faculty of Medicine, University of Helsinki, Helsinki, Finland; 6 Department of Clinical Pharmacology and HUSLab, University of Helsinki, Helsinki, Finland

**Keywords:** Methylprednisolone, Neonate, Infant, Cardiac surgery, Pharmacokinetics

## Abstract

**Purpose:**

Methylprednisolone is used during paediatric cardiac surgery with cardiopulmonary bypass to attenuate the systemic inflammatory response. However, developmental differences in drug disposition and cardiopulmonary bypass–related factors contribute to pharmacokinetic variability in this population. We aimed to develop a population pharmacokinetic model of methylprednisolone in neonates and paediatric patients undergoing cardiac surgery with cardiopulmonary bypass and to identify factors associated with its pharmacokinetic variability.

**Methods:**

Data were pooled from four prospective clinical studies including 93 patients aged 1 to 574 days undergoing cardiac surgery with cardiopulmonary bypass. Methylprednisolone was administered as an intravenous bolus after anaesthesia induction or into the cardiopulmonary bypass circuit at 5 or 30 mg/kg. Plasma concentrations were measured perioperatively. A nonlinear mixed-effects model was developed using NONMEM. Covariate effects were evaluated. Model evaluation included goodness-of-fit plots, normalized prediction distribution errors, sampling importance resampling, and prediction-corrected visual predictive checks.

**Results:**

A one-compartment model with first-order elimination adequately described the data. The final model identified body weight, an empirical postmenstrual-age effect on clearance, and operation-group differences in the apparent volume of distribution as significant determinants of methylprednisolone pharmacokinetics. Interindividual variability was estimated for clearance (48.1%) and volume of distribution (19.9%). Model evaluation supported the predictive performance of the final model.

**Conclusion:**

We developed a population pharmacokinetic model for methylprednisolone in paediatric patients undergoing cardiac surgery with cardiopulmonary bypass. The model identifies body weight, postmenstrual age and operation group as determinants of pharmacokinetic variability. It provides a descriptive framework for characterizing methylprednisolone disposition in this heterogeneous population.

**Trial registration number and date:**

EudraCT 2008-007413-76 2008-12-23.

**Supplementary Information:**

The online version contains supplementary material available at https://doi.org/10.1007/s00228-026-04168-7.

## Introduction

Cardiopulmonary bypass (CPB) during paediatric cardiac surgery provokes a potent systemic inflammatory response characterized by cytokine release, complement activation, endothelial injury, and organ dysfunction [1]. In this setting, corticosteroids such as methylprednisolone (MP) have been used for decades as adjunctive agents to attenuate CPB-induced inflammation. MP is a synthetic glucocorticoid that modulates immune function through genomic and non-genomic mechanisms, notably reducing proinflammatory cytokines such as IL-6 while enhancing anti-inflammatory mediators such as IL-10 [2]. However, the benefit–risk balance of MP in paediatric cardiac surgery remains controversial.

Despite extensive clinical use, there is no consensus on optimal MP dosing or timing in neonates and children undergoing cardiac surgery. A systematic review and meta-analysis by Chen et al. demonstrated that while perioperative corticosteroids, including MP and dexamethasone, reduced hospital length of stay, they did not consistently reduce mortality in paediatric cardiac surgery patients [3]. A broader meta-analysis including both paediatric and nonelderly adult populations [4] found that corticosteroids were associated with a 31% relative reduction in mortality and decreased vasoactive inotropic scores, but no significant difference in complications such as acute kidney injury or infection. These discrepancies may stem from interindividual variability, differing dosing regimens, and developmental pharmacokinetics that are not well captured in traditional trial designs.

Recent efforts have sought to clarify the pharmacological profile of MP in neonates undergoing CPB. In a pivotal study, Hornik et al. [5] used population pharmacokinetic/pharmacodynamic (popPK/PD) modelling to relate MP exposure to IL-6 and IL-10 dynamics, showing that 10 mg/kg achieved maximal biomarker modulation, and that increasing the dose to 30 mg/kg offered no additional benefit. However, the study was limited to neonates within the first 30 days of life, precluding investigation of age-dependent clearance maturation.

Further insights into MP disposition during CPB were provided by van Saet et al. [6], who observed a substantial drop in MP plasma concentrations following CPB initiation and demonstrated a longer elimination half-life in neonates compared to older children. These findings underscore the need for weight- and age-adjusted dosing schemes and highlight the impact of CPB-related dilutional effects on steroid exposure.

Most recently, the STRESS trial [7], a multicentre randomized controlled trial in 1200 infants; found no significant improvement in composite clinical outcomes with MP (30 mg/kg), although secondary analyses suggested modest benefit. Importantly, MP increased the risk of hyperglycaemia requiring insulin. This large trial highlights the uncertainty around MP efficacy and the potential for harm in low-risk patients.

Given the variable findings from clinical trials and limited paediatric PK data, further pharmacometric studies are essential. The aim of our study was to develop a robust population pharmacokinetic model for methylprednisolone in neonates and paediatric patients undergoing cardiac surgery, quantify variability in drug exposure, and explore covariates influencing disposition.

## Methods

### Ethics and informed consent

All study protocols were assessed by the Ethics Committee of Helsinki University Central Hospital and the Finnish Medicines Agency. The studies were approved by the Institutional Review Board of the Helsinki University Central Hospital. Individual studies were registered in the European Clinical Trials Database (EudraCT 2008-007413-76) maintained by the European Medicines Agency. Written informed consent was obtained from parents or legal guardian before the patients were enrolled in the studies. All studies were in accordance with both the ethical standards of the institutional research committee and the 1964 Declaration of Helsinki and its later amendments.

### Studies and perioperative care

We pooled the data from 93 children enrolled in four previous studies [8–11], which evaluated the effects of methylprednisolone (MP) in children undergoing open-heart surgery with CPB. Summary of the individual studies are presented in Table 1.

**Table 1:** Summary of the original clinical trials and the clinical characteristics of the studied patients based on the type of surgery

Parameter	All	Neonates	TOF	VSD/AVSD	GLENN
n	93	20	28	30	15
sex (F/M)	53/40	12/8	15/13	16/14	10/5
Postnatal age (d)	140 (1-574)	7 (1–27)	164 (71–311)	144 (98–574)	180 (94–371)
Postmenstrual age (d)^a^	424 (270–854)	290 (270–316)	439 (351–591)	425 (378–854)	460 (374–651)
Body weight (kg)	5.8 (1.9)	3.6 (0.41)	7.0 (1.9)	5.8 (1.5)	6.7 (1.1)
Methylprednisolone dose (mg)	159 (58)	107 (12)	160 (75)	174 (44)	199 (34)
Dosing scheme (mg/kg)^b^	-	30	5 or 30	30	30
Sampling period (min, range)	23–758	34–563	67–615	79–758	23–736
CPB time (min)	97 (53)	168 (43)	97 (29)	65 (27)	68 (47)
No. of samples	279^c^	3^c^	3	3	3
Reference	-	Keski-Nisula et al., 2013	Keski-Nisula et al., 2016	Keski-Nisula et al., 2015	Keski-Nisula et al., 2020

Unless otherwise stated, the values are expressed as mean and SD except for ages, which are reported as median (range)

TOF, tetralogy of Fallot; VSD/AVSD, ventricular or atrioventricular septal defect; GLENN, procedure for patients with tricuspid atresia; F, female; M, male

^a^For TOF, VSD/AVSD and Glenn-groups, a gestational age of 280 days was assumed when calculating postmenstrual age

^b^ Intravenous dose at anaesthesia induction. For VSD/AVSD-group, methylprednisolone dose was randomized to be administered either intravenously (i.v.) at anaesthesia induction or to priming solute in the cardiopulmonary bypass-circuit (*n* = 15 in both groups)

^c^Total no. of samples, and Median no. of samples/patient

Balanced anaesthesia was induced with propofol or S-ketamine, sufentanil and pancuronium and maintained with sevoflurane. Myocardial protection and CPB were performed as described previously [12]. MP was administered using three different protocols, according to study cohort:

- Neonatal patients (Neonates-study) and patients with tricuspid atresia (Glenn procedure-study) received a single intravenous bolus of MP 30 mg/kg immediately after anaesthesia induction and arterial line placement.
- Patients undergoing total correction of tetralogy of Fallot (TOF-study) were randomized to receive an intravenous bolus of MP 5 or 30 mg/kg immediately after anaesthesia induction.
- Patients with ventricular or atrioventricular septal defect (VSD/AVSD-study) were randomized to receive an MP bolus of 30 mg/kg either intravenously after induction of anaesthesia or into the CPB circuit.

Intraoperative management, inotrope therapy and postoperative care have been previously described [8–11].

### Blood samples and drug analyses

Arterial blood samples (5 ml each) were collected into sodium citrate containing tubes at three time points: 30 min after initiation of CPB, 5 min after administration of protamine and 6 h after cessation of CPB. Plasma was separated immediately by centrifugation and was stored at − 70^ₒ^C until analysis. Total plasma concentration of MP was determined by using a previously published high-performance liquid chromatography–electrospray–tandem mass spectrometry method [13]. The lower limit of MP quantification was 2 ng/ml, and the interday coefficient of variation was less than 10%.

### Pharmacometric analysis

The methylprednisolone pharmacokinetic model was developed in stages comprising evaluation of the structural model, interindividual variability, covariate relationships, and residual variability, followed by model evaluation. Doses administered directly intravenously or into the cardiopulmonary bypass circuit were treated as intravenous bolus inputs. Time was expressed in minutes, dose in milligrams, plasma concentration in ng/ml, clearance in l/min, and volume of distribution in litres.

The structural model was developed first, and one- and two-compartmental models with first-order elimination were tested to describe the concentration-time data. Between-subject variability of the pharmacokinetic parameters was estimated assuming a log-normal distribution:

$$\theta_i=\theta\cdot e^{\eta_i}$$

in which $\:{\theta\:}_{i}$ is the individual parameter value for $\:{i}^{th}$ individual, $\:\theta\:$ is the typical value of this parameter in the population and $\:{\eta\:}_{i}$ is a random variable with mean of zero and variance $\:{\omega\:}_{\eta\:}^{2}$. For the residual errors, describing intra-individual variability, a proportional error model was used:

$$\:{y}_{ij}={ipred}_{ij}\left(1+{\epsilon\:}_{prop}\right)$$

where $\:{y}_{ij}$ is the $\:{j}^{th}$ measured observation of the $\:{i}^{th}$ individual, $\:{ipred}_{ij}$ the corresponding individual prediction defined by the structural model, and $\:{\epsilon\:}_{prop}$ is proportional random variable assumed to be normally distributed with mean of zero and standard deviation of $\:\sigma\:$. Separate residual error models were estimated for each study.

Data were modelled with non-linear mixed-effects modelling using NONMEM (Version 7.5.0, ICON Development solutions, Ellicot City, MD, USA). Perl-Speaks-NONMEM (PsN) toolkit was used to aid the model development, execution and evaluation [14]. In addition, the R programming language [15] and RStudio [16] were used for exploratory data analysis, statistical analysis and graphing.

### Covariate analysis

Body weight, postmenstrual age (PMA), neonatal status, type of operation, route of methylprednisolone administration, duration of cardiopulmonary bypass, lowest intraoperative temperature, and duration of circulatory arrest were evaluated as potential covariates. Candidate covariate relationships were assessed sequentially by comparing nested models and considering changes in the objective function value, parameter estimates and precision, shrinkage, biological plausibility, and diagnostic plots.

Several models for weight and age have been proposed to scale clearance (CL) and volume (V) in children [17–20]. Body weight was incorporated using fixed allometric scaling, with exponents of 0.75 for clearance and 1.0 for volume of distribution. Body weight was centred at the median value of the study population (5.7 kg). A continuous empirical PMA effect on clearance was evaluated using a centred power relationship:

$$\:{CL}_i={CL}_{TV}\cdot\:\left(\frac{{WT}_i}{{WT}_{med}}\right)^{0.75}{\cdot\:\left(\frac{{PMA}_i}{{PMA}_{med}}\right)}^{{\theta\:}_{PMA}}$$

where $\:C{L}_{i}$is the typical clearance for an individual with body weight $\:W{T}_{i}$and postmenstrual age $\:PM{A}_{i}$, $\:{\theta\:}_{CL}$is the typical clearance at the median body weight ($\:{WT}_{med}$) and PMA $\:{(PMA}_{med})$, and $\:{\theta\:}_{PMA}$is the estimated empirical PMA exponent. PMA was expressed in days and centred at the median value of 424 days.

Other continuous covariates, including postmenstrual age, duration of cardiopulmonary bypass, lowest intraoperative temperature, and duration of circulatory arrest, were evaluated as centred continuous covariates on pharmacokinetic parameters using linear or power relationships, as appropriate. Effects of categorical covariates on pharmacokinetic parameters were evaluated using multiplicative log-scale relationships.

### Model evaluation

Model development was based on likelihood ratio test comparing differences in minimum OFV, parameter precision, error estimates and shrinkage values. Furthermore, the choice between models was based on the recently proposed goodness-of-fit plots [21].

The robustness of the final model was evaluated using a sampling importance resampling (SIR) procedure [22]. SIR was performed in Perl-speaks-NONMEM using three sequential iterations with increasing proposal sample sizes and resample sizes to obtain stable estimates of parameter uncertainty. Median parameter estimates and 95% confidence intervals were derived from the final SIR iteration. Normalized prediction distribution errors (NPDEs) based on 1000 simulations were calculated in NONMEM [23], and their distribution plotted against population-predicted concentrations, time and selected covariates. Prediction-corrected visual predictive checks (pcVPCs) were used to assess the predictive performance of the final model [24].

## Results

**Fig. 1:**
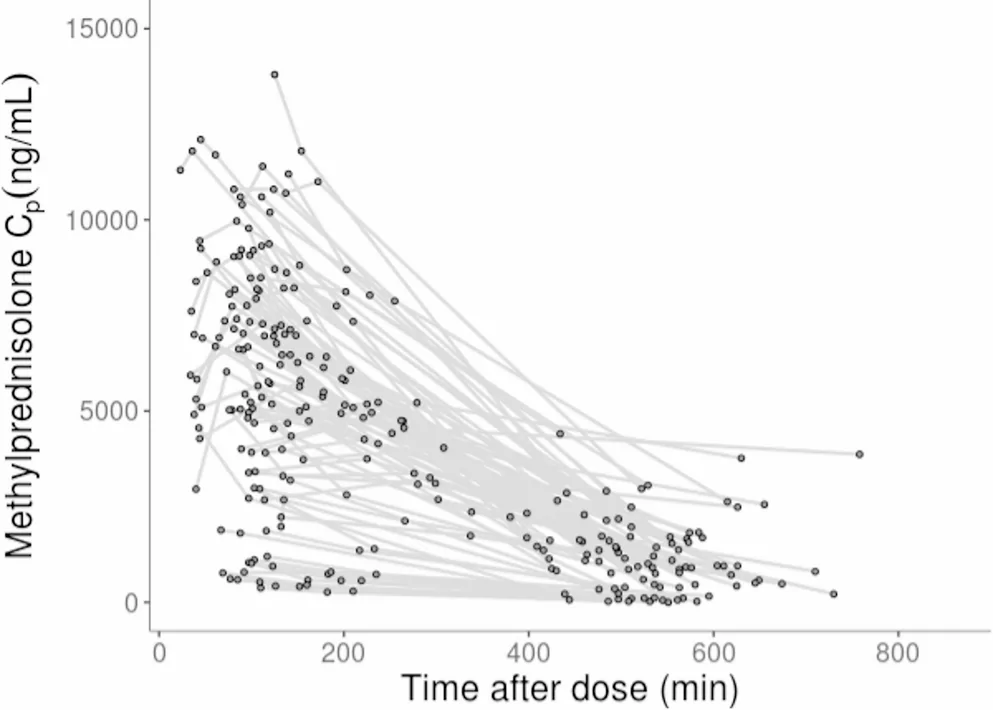
Concentration-time profile from the observed data

Details of the individual studies, and descriptive characteristics for the study population of 93 paediatric cardiac surgery patients are summarised in Table 1. MP drug concentrations were available from 279 plasma samples, of which one was below the lower limit of quantification and was excluded from the dataset. Consequently, 278 plasma samples (median three samples per patient) were included in the population pharmacokinetic analysis. The observed plasma concentrations are shown in Fig. 1.

### Population pharmacokinetic model

Based on the concentration–time plots of the original data, one- and two-compartment models were tested, since it seemed that the elimination phase might show a two-phase behaviour. One- and two-compartment structural models were evaluated. Although the two-compartment model reduced the objective function value by approximately 25 units compared with the one-compartment model, improvements in diagnostics (GOF, NPDE and pcVPC) were modest. Therefore, the one-compartment model was selected as the structural model according to the principle of parsimony.

Body weight was incorporated using fixed allometric scaling. Among the investigated categorical covariates, operation type significantly improved the model when included on volume of distribution (ΔOFV = 43.5), whereas modelling operation type on clearance resulted in severe ill-conditioning despite a smaller OFV improvement and was therefore rejected.

Continuous covariates representing lowest intraoperative temperature and duration of circulatory arrest did not improve model performance and were not retained. Likewise, removal of the neonatal indicator resulted in only a small increase in objective function value (ΔOFV = 3.18) and the simpler model was therefore accepted. Addition of a continuous empirical postmenstrual age (PMA) effect on clearance further improved the model (ΔOFV = − 5.69). Subsequent inclusion of interindividual variability on volume of distribution resulted in an additional reduction of 3.98 objective function value units. The 95% SIR confidence intervals for the PMA exponent and the interindividual variability on volume excluded zero, supporting retention of both model components. The resulting model was selected as the final population pharmacokinetic model.

### Final population pharmacokinetic parameter estimates

Final population pharmacokinetic parameter estimates are presented in Table 2. Typical clearance and volume of distribution for a 5.7-kg patient with a PMA of 424 days were estimated as 4.57 L/h (95% CI 4.15–5.06 L/h) and 27.1 L (95% CI 23.1–31.6 L), respectively. The estimated PMA exponent describing the empirical maturation effect on clearance was 0.503. Operation type influenced the volume of distribution, with all surgical groups showing lower typical volumes than the reference group (OPER 0). Interindividual variability was estimated for both clearance and volume of distribution, corresponding to coefficients of variation of 48.1% (95% CI 39.9–59.6%) and 19.9% (95% CI 12.4–26.5%), respectively.

**Table 2:** Pharmacokinetic parameter estimates for the final population pharmacokinetic model of methylprednisolone in paediatric patients undergoing cardiac surgery

Parameter	NONMEM estimate^1^	RSE (%)	SIR-based 95% confidence interval	Derived value or interpretation
Structural parameters				
THETA(1): Typical CL (L/min)	0.0761	6.38	0.0692–0.0843	Typical CL at 5.7 kg and PMA 424 days.
THETA(2): Typical V (L)	27.1	7.90	23.1–31.6	Typical V at 5.7 kg in OPER 0.
Covariate effects				
THETA(3): Empirical PMA exponent on CL	0.503	45.0	0.0881–0.929	Empirical PMA exponent on CL
THETA(4): Log V effect, OPER 1 vs. 0	−0.404	36.2	−0.621–−0.155	V ratio 0.668 vs. OPER 0 (SIR-derived ratio interval 0.537–0.857)^2,3^
THETA(5): Log V effect, OPER 2 vs. 0	−0.475	19.7	−0.660–−0.295	V ratio 0.622 vs. OPER 0 (SIR-derived ratio interval 0.517–0.744)
THETA(6): Log V effect, OPER 3 vs. 0	−0.884	12.8	−1.10–−0.672	V ratio 0.413 vs. OPER 0 (SIR-derived ratio interval 0.334–0.511)
Interindividual variability (IIV)				
OMEGA(1,1): IIV variance, CL	0.208	26.0	0.148–0.304	IIV CV 48.1% (SIR-derived interval 39.9–59.6%)^3^
OMEGA(2,2): IIV variance, V	0.0388	51.6	0.0152–0.0678	IIV CV 19.9% (SIR-derived interval 12.4–26.5%)
Residual variability				
SIGMA(1,1): Proportional residual variance, OPER 0	0.0851	19.9	0.0556–0.125	Residual SD 29.2% (SIR-derived interval 23.6–35.4%)^3^
SIGMA(2,2): Proportional residual variance, OPER 1	0.179	16.6	0.126–0.248	Residual SD 42.3% (SIR-derived interval 35.5–49.8%)
SIGMA(3,3): Proportional residual variance, OPER 2	0.0428	24.0	0.0284–0.0665	Residual SD 20.7% (SIR-derived interval 16.8–25.8%)
SIGMA(4,4): Proportional residual variance, OPER 3	0.0197	63.9	0.00869–0.0452	Residual SD 14.0% (SIR-derived interval 9.32–21.3%)
Fixed scaling parameters				
Allometric body-weight exponent for CL	0.750 (fixed)	—	—	Fixed body-weight scaling exponent for CL
Allometric body-weight exponent for V	1.00 (fixed)	—	—	Fixed body-weight scaling exponent for V

CI, confidence interval; CL, clearance; CV, coefficient of variation; OPER, operation group: 0, neonates (reference); 1, tetralogy of Fallot; 2, ventricular or atrioventricular septal defect; 3, Glenn procedure; PMA, postmenstrual age; RSE, relative standard error; SIR, sampling importance resampling; V, volume of distribution

^1^Point estimates are final NONMEM estimates. RSE values are derived from the successful covariance-step standard errors. The 95% confidence intervals are the final SIR 2.5th and 97.5th percentiles. RSE and the principal SIR interval refer to the original estimated parameter scale

^2^Derived quantities: OPER V ratio = exp(θ_OPER_); IIV CV% = 100 × √[exp(ω) − 1]; residual SD% = 100 × √σ²

^3^Derived ratios, CV values, and residual SD values are interpretive and do not have separate covariance-derived RSE values in this table

The covariance step completed successfully, and all parameter estimates were obtained with acceptable precision. Sampling importance resampling (SIR) confirmed the robustness of the final model, with SIR median estimates closely matching the NONMEM estimates and 95% confidence intervals supporting the inclusion of the PMA effect on clearance and operation group effects on volume of distribution. Complete parameter estimates, relative standard errors, and SIR-derived 95% confidence intervals are presented in Table 2.

### Model evaluation

**Fig. 2:**
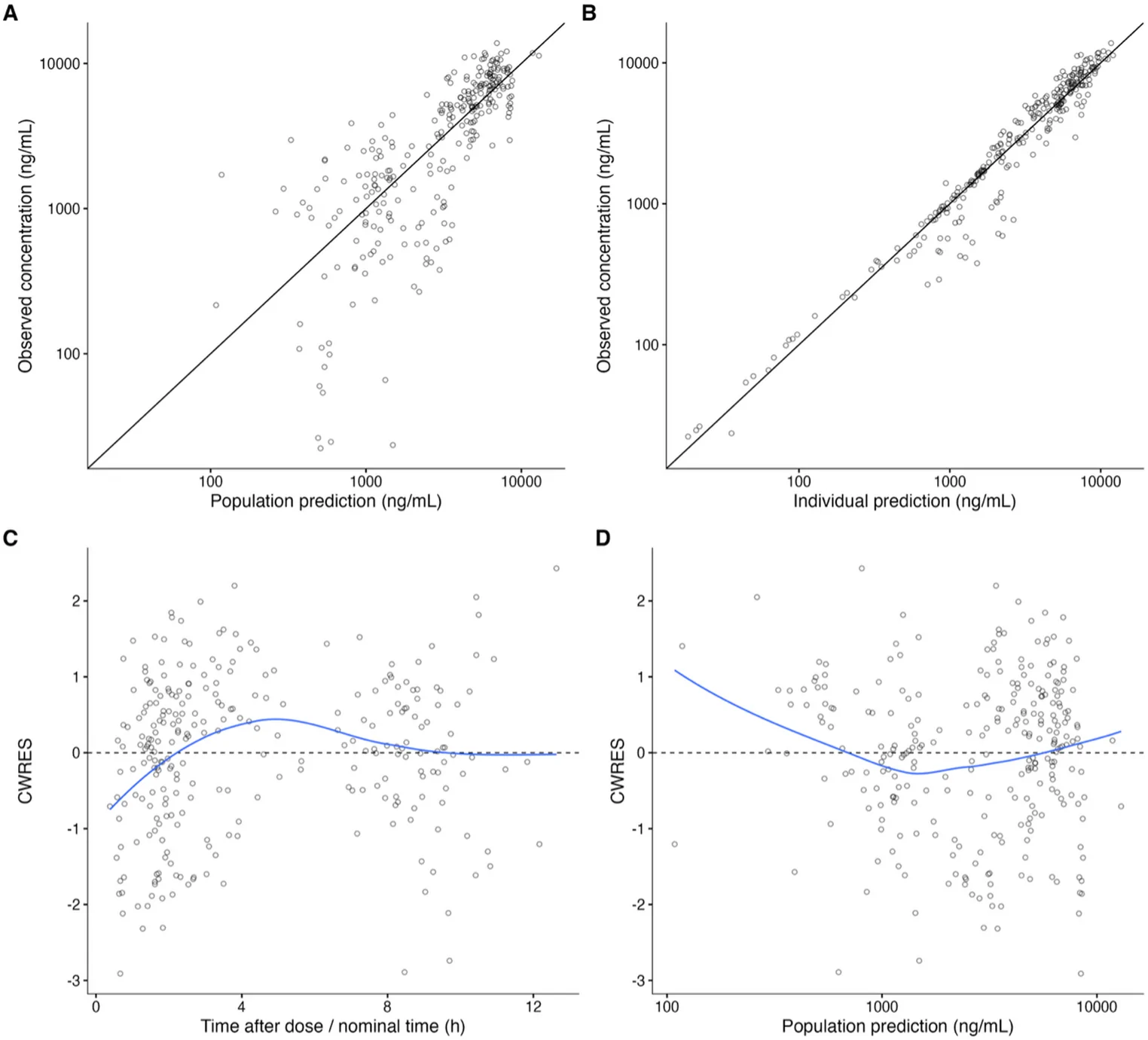
Goodness-of-fit plots. Observed versus population-predicted and individual-predicted methylprednisolone concentrations, together with conditional weighted residuals (CWRES) versus time and population-predicted concentrations. The solid line represents the line of identity in the observed-versus-predicted plots, and the horizontal line indicates zero residuals in the CWRES plots. The blue curve is a locally weighted scatterplot smoother (LOESS) to highlight potential trends

**Fig. 3:**
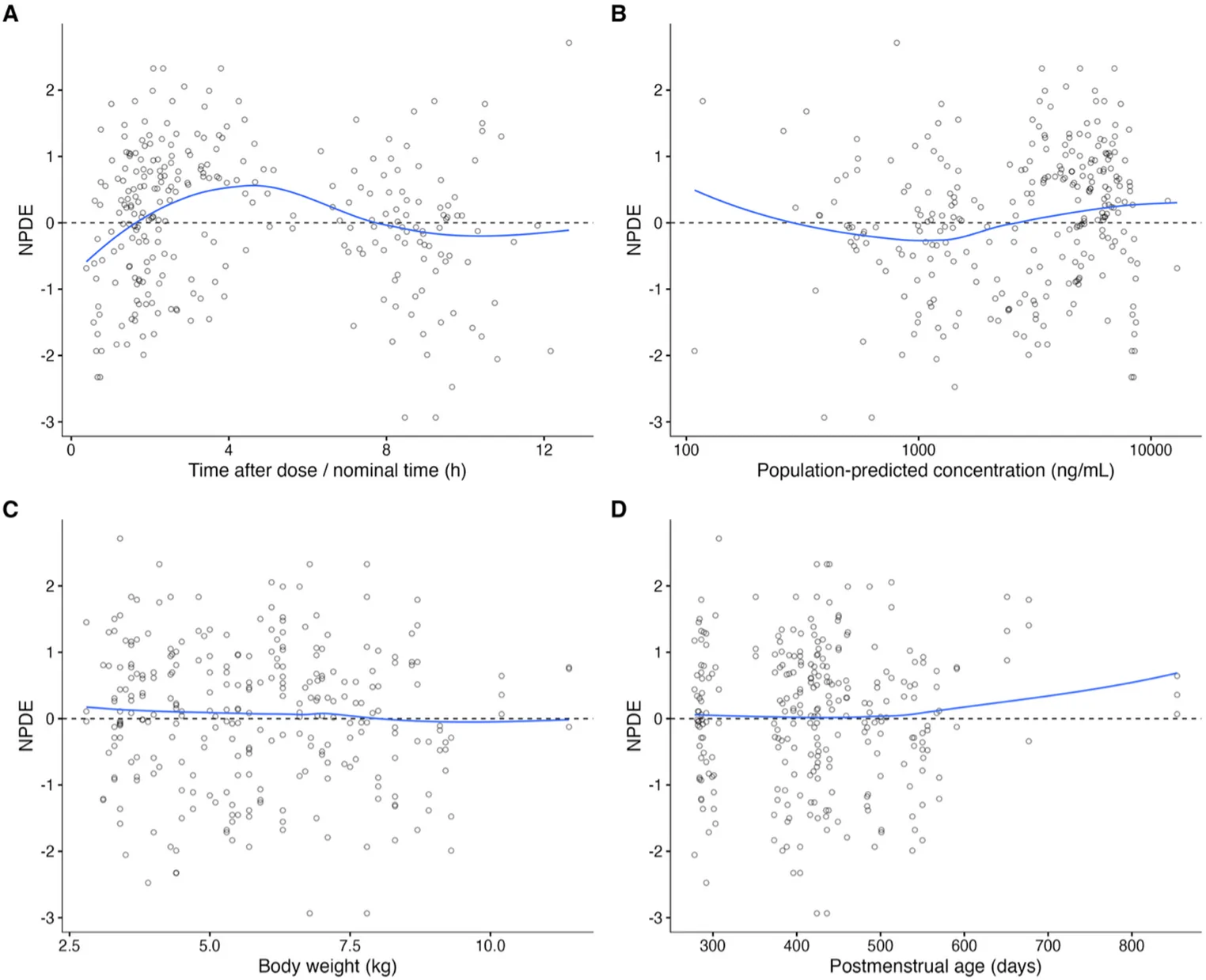
Normalized prediction distribution error (NPDE) diagnostics. Scatter plots of NPDE versus (**A**) time, (**B**) population-predicted concentrations, (**C**) body weight, and (**D**) postmenstrual age (PMA). The blue line represents a locally weighted scatterplot smoother (LOESS)

Goodness-of-fit plots demonstrated adequate agreement between the observed and model-predicted methylprednisolone concentrations (Fig. 2). Conditional weighted residuals were symmetrically distributed around zero without major systematic trends over time or population-predicted concentrations. NPDE diagnostics showed no substantial deviations from the expected distribution and no clinically relevant relationships with time, postnatal age or body weight (Fig. 3).

**Fig. 4:**
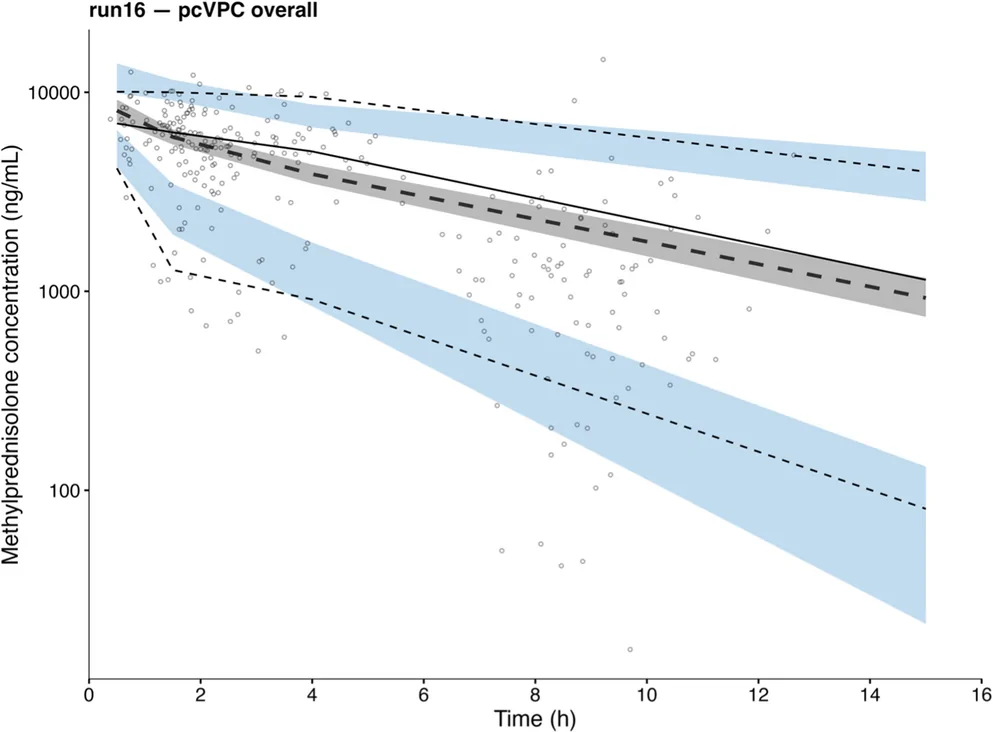
Prediction-corrected visual predictive check (pcVPC) based on 1,000 simulations with the final population pharmacokinetic model. Grey dots represent individual observations. The black solid and dashed lines represent the observed median and the 5th and 95th percentiles of the observed plasma concentrations, respectively. The blue shaded area denotes the simulation-based 95% confidence interval for the predictions. Time refers to time after the methylprednisolone administration

Prediction-corrected visual predictive checks demonstrated generally good agreement between the observed and simulated concentration–time profiles overall and across operation groups (Fig. 4), although minor discrepancies were observed during the early post-dose period. Supplementary Figure S1 presents prediction-corrected visual predictive checks stratified by study (operation group). These showed generally adequate agreement between the observed and simulated concentration distributions across the four study cohorts (Supplementary Figure S1). Overall, the diagnostic evaluation supported the adequacy of the final population pharmacokinetic model.

## Discussion

Our study presents a population pharmacokinetic model for methylprednisolone in neonates and paediatric patients undergoing cardiac surgery with cardiopulmonary bypass (CPB). The model was developed using pooled data from four prospective studies with distinct dosing strategies and age distributions. By characterizing methylprednisolone disposition across a broader paediatric age spectrum than previous studies, our findings provide new information on interindividual variability and the empirical relationships between clearance, postmenstrual age, body weight and operation group in this population.

### Developmental pharmacokinetics and interindividual variability

The observed association between postmenstrual age and methylprednisolone clearance is consistent with current understanding of developmental pharmacokinetics during infancy and childhood. Maturational changes in hepatic enzyme activity, plasma protein binding, and organ perfusion are all expected to contribute to age-dependent variability in corticosteroid disposition [25]. Although our final model retained postmenstrual age as an empirical continuous covariate rather than a mechanistic maturation function, these findings support the concept that developmental maturation remains an important contributor of methylprednisolone pharmacokinetics in this population.

Van Saet and colleagues reported an acute decrease in methylprednisolone plasma concentrations immediately after initiation of CPB, which was considered consistent with haemodilution, altered protein binding and sequestration within the CPB circuit [6]. Similar concentration changes were observed in our dataset, supporting the concept that CPB substantially influences early methylprednisolone disposition. Nevertheless, appreciable interindividual variability remained after accounting for body weight, postmenstrual age and operation group, suggesting that additional physiological and perioperative factors influencing methylprednisolone disposition were not captured in the present dataset. Although our sparse sampling did not permit detailed characterization of these processes, they probably contributed to the remaining variability observed in this population.

### Comparison with previous PK/PD models

Our findings are broadly consistent with previous pharmacokinetic studies of methylprednisolone in paediatric cardiac surgery, while differing in model structure and study design. Hornik et al. developed a population pharmacokinetic/pharmacodynamic model in neonates undergoing cardiopulmonary bypass that incorporated developmental maturation and linked methylprednisolone exposure with suppression of interleukin-6 concentrations [5]. In contrast, our analysis included a broader paediatric population spanning neonates to older children undergoing different cardiac procedures and focused on characterising population pharmacokinetics rather than pharmacodynamic effects.

Despite these differences, both studies support the importance of developmental factors in explaining methylprednisolone pharmacokinetic variability. Whereas Hornik et al. described developmental maturation using a mechanistic maturation function [5], our final model retained postmenstrual age as an empirical continuous covariate on clearance after fixed allometric scaling. Given the sparse sampling design and the strong correlation between developmental covariates in our dataset, this parsimonious approach was considered more appropriate for the present analysis.

The broader age range and pooled study design also allowed evaluation of differences between operation groups. These operation groups represent clinically distinct patient populations undergoing different procedures at different stages of development, and therefore inherently combine developmental, anatomical and perioperative characteristics. Consequently, the observed operation-group effects should be interpreted as empirical descriptors of the pooled data rather than as isolated effects of the surgical procedure itself.

### Clinical relevance and integration with recent trials

The role of perioperative methylprednisolone in paediatric cardiac surgery remains an area of active clinical interest. The STRESS trial and other recent randomized studies have reported variable effects of corticosteroid therapy on clinical outcomes, highlighting the complexity of balancing the anti-inflammatory benefits of corticosteroids against their potential adverse effects [3, 7, 26]. More recently, Losiggio et al. concluded in a meta-analysis of randomized trials that perioperative corticosteroid therapy was associated with potential survival benefit and improvement in selected clinical outcomes in paediatric and non-elderly patients undergoing cardiac surgery with cardiopulmonary bypass [4]. These findings reinforce the continued clinical relevance of methylprednisolone and provide further justification for improving our understanding of its pharmacokinetics in this setting.

Although the present model was not designed to evaluate exposure-response relationships, it demonstrates substantial pharmacokinetic variability despite accounting for body weight, postmenstrual age and operation group. The model therefore provides a quantitative framework for future pharmacokinetic-pharmacodynamic studies investigating whether variability in methylprednisolone exposure contributes to the heterogeneous clinical findings reported in recent trials.

### Strengths and limitations

A major strength of our study is the integration of data across four prospective studies with harmonized protocols and rigorous MP quantification. This enabled the inclusion of patients across a broad paediatric age range undergoing different cardiac procedures and provided a heterogeneous dataset for population pharmacokinetic modelling. Another strength is the comprehensive evaluation of the final model.

However, limitations remain. First, our study is observational and does not directly link MP exposure to clinical outcomes such as ICU stay or inflammatory biomarkers. Second, pharmacokinetic sampling was sparse, with most patients contributing three concentration measurements. Consequently, the data supported a parsimonious descriptive population model but limited characterization of more complex distributional processes and mechanistic maturation. The postmenstrual-age effect retained in the final model should therefore be interpreted as an empirical covariate relationship rather than a validated mechanistic maturation function. Finally, the scope of the present study was limited to descriptive population pharmacokinetic modelling, and therefore dosing simulations and exposure-target analyses were beyond its objectives.

### Future Directions

Population pharmacokinetic studies in paediatric cardiac surgery are inherently challenging, as opportunities for informative pharmacokinetic sampling are limited in this vulnerable patient population. Future studies integrating pharmacokinetic, pharmacodynamic and clinical outcome data could better define the exposure–response relationship of methylprednisolone in this setting. Richer pharmacokinetic sampling would also allow evaluation of mechanistic maturation models and more detailed characterization of cardiopulmonary bypass–related effects on methylprednisolone disposition. External evaluation in independent cohorts will be important to establish the generalizability of the present model and support future pharmacokinetic–pharmacodynamic investigations.

## Conclusion

We developed and internally evaluated a population pharmacokinetic model for methylprednisolone in neonates and paediatric patients undergoing cardiac surgery with cardiopulmonary bypass. The final model identified body weight, an empirical postmenstrual-age effect on clearance, and operation-group differences in the apparent volume of distribution as important determinants of methylprednisolone pharmacokinetics. Substantial unexplained interindividual variability remained. The model provides a descriptive framework for characterizing methylprednisolone disposition in a heterogeneous paediatric cardiac surgery population.

## Supplementary Information

Below is the link to the electronic supplementary material.

Supplementary Material 1 (PDF 401 KB)

## Data Availability

The data that support the findings of this study are not openly available due to reasons of sensitivity and are available from the corresponding author upon reasonable request. Data are located in controlled access data storage at University of Turku.

## Declarations

### Trial registration number and date

EudraCT 2008-007413-76 2008-12-23.
