# Supplementary material for: Population pharmacokinetics of methylprednisolone in neonates and paediatric patients undergoing cardiac surgery with cardiopulmonary bypass
Source: Eur J Clin Pharmacol. 2026 Aug 18;82(9):235. doi: 10.1007/s00228-026-04168-7 (PMC13486032; doi:10.1007/s00228-026-04168-7)

Population Pharmacokinetics of Methylprednisolone in Neonates and Paediatric Patients Undergoing Cardiac Surgery with Cardiopulmonary Bypass

European Journal of Clinical Pharmacology

Matias Rantanen, Juho Keski-Nisula, Klaus T. Olkkola, Pertti J. Neuvonen, Eero Pesonen, Pertti Suominen, Teijo I. Saari

Corresponding author:

Teijo I. Saari, Department of Anaesthesiology and Intensive Care, University of Turku, Kiinamyllynkatu 4-8, 20521 Turku, Finland. Tel.: +358-2-313-296. E-mail: teisaa@utu.fi

<u>SUPPLEMENTARY MATERIAL</u>

Supplementary Figure S1. Prediction-corrected visual predictive checks (pcVPCs) based on 1,000 simulations with the final population pharmacokinetic model stratified by operation group (operation group, OPER). Grey dots represent individual observations. The black solid and dashed lines represent the observed median and the 5th and 95th percentiles of the observed plasma concentrations, respectively. The blue shaded area denotes the simulation-based 95% confidence interval for the predictions. Time refers to time after methylprednisolone administration. Panels show (A) neonates (OPER 0), (B) tetralogy of Fallot (OPER 1), (C) ventricular or atrioventricular septal defect (OPER 2), and (D) bidirectional Glenn procedure (OPER 3).

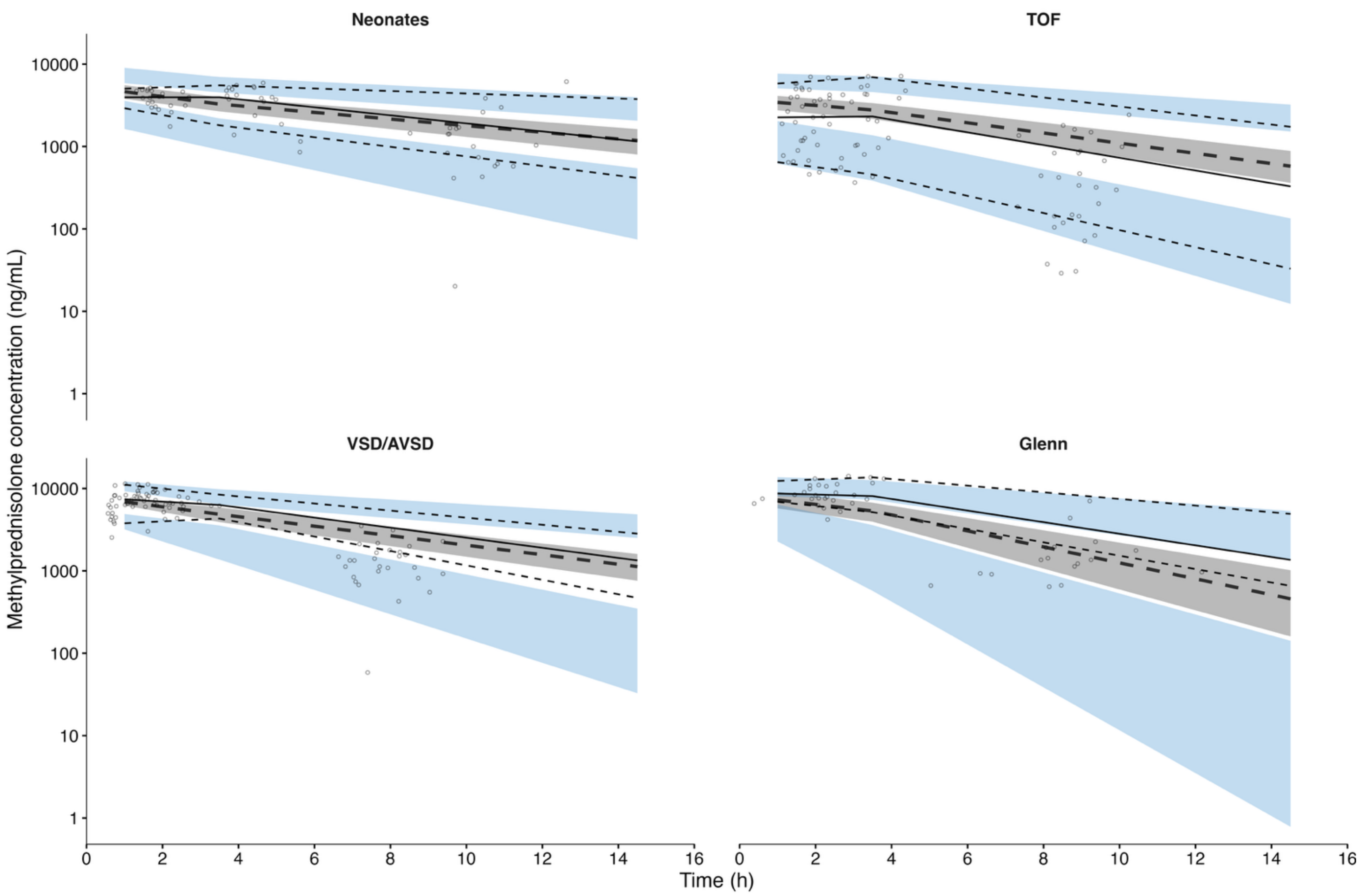

Supplement: Supplementary file 1 [file 228_2026_4168_MOESM1_ESM.pdf]
